# In vivo molecular skin fluorescence imaging for noninvasive assessment of atypical nevi and melanoma: A validation trial

**DOI:** 10.1016/j.jdin.2025.11.010

**Published:** 2025-11-26

**Authors:** Douglas Grossman, Elizabeth Berry, Justin M. Ko, Raja Sivamani, Serena Mraz, Sunil S. Dhawan, Kevin Manbeck, Amit Shachaf, Shamika Majmudar, Catherine Shachaf, Sancy Leachman

**Affiliations:** a Department of Dermatology, University of Utah Health Sciences Center, Salt Lake City, Utah; b Huntsman Cancer Institute, Salt Lake City, Utah; c Department of Dermatology, Oregon Health & Science University, Portland, Oregon; d Department of Dermatology, Stanford University School of Medicine, Stanford, California; e Pacific Skin Institute, Sacramento, California; f Solano Dermatology Associates, Vallejo, California; g Center for Dermatology Cosmetic and Laser Surgery, Fremont, California; h Orlucent Inc., Los Gatos, California

**Keywords:** clinical study, fluorescence imaging, melanoma, nevus, tissue remodeling, αvβ3 integrin

## Abstract

**Background:**

Current visual assessments and assistive technologies for melanoma detection primarily focus on detecting morphological changes, often requiring invasive biopsies for confirmation.

**Objective:**

To determine the efficacy of skin fluorescent imaging (SFI), a novel noninvasive technology for detection of αvβ3 integrin in the tumor microenvironment, for discrimination of benign from malignant melanocytic lesions.

**Methods:**

A prospective validation trial evaluated 240 clinically suspicious cutaneous pigmented lesions prior to biopsy in 6 academic and community dermatology clinics in California, Utah, and Oregon.

**Results:**

The distribution of lesions included 99 (41%) lesions without dysplasia, 59 (25%) nevi with low-grade dysplasia, 51 (21%) nevi with high-grade dysplasia, 20 (8.3%) melanoma in situ, and 11 invasive melanomas (4.6%). SFI cutoff scores of 5 and 7 demonstrated sensitivities of 93% and 87% and specificities of 77% and 91%, respectively, for discrimination of lesions with no or low-grade dysplasia from those with high-grade dysplasia or melanomas. The area under the receiver operating characteristic curve was 0.907 (95% CI 0.864-0.951, *P* <.0001).

**Limitations:**

This was a single-arm trial.

**Conclusions:**

SFI demonstrated high sensitivity and specificity for discriminating low-risk from high-risk lesions, representing a promising approach to enhance evaluation of clinically concerning pigmented lesions and reduce unnecessary biopsies.

## Graphical abstract

**Figure:**
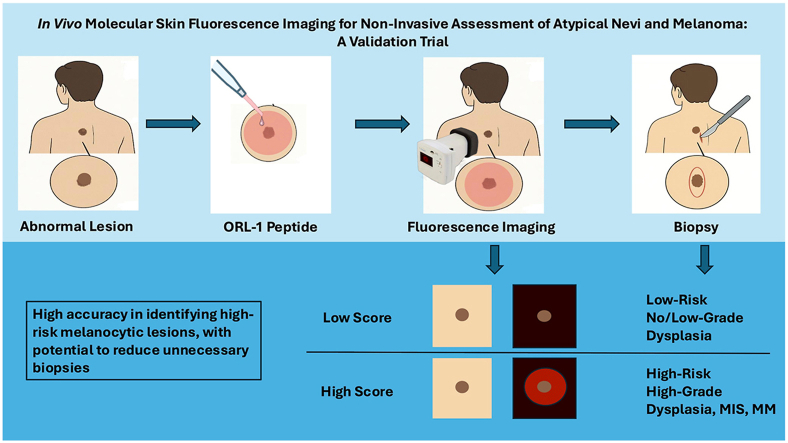

**Capsule Summary**

• Skin fluorescent imaging (SFI) is a novel noninvasive technology for detection of αvβ3 integrins in the tumor microenvironment.
• In a validation study of 240 clinically suspicious pigmented lesions, SFI demonstrated high sensitivity and specificity for discrimination of lesions with no or low-grade dysplasia from those with high-grade dysplasia or melanoma.

## Introduction

Skin biopsy remains the gold standard approach to diagnose melanoma, yet a systematic review of 46 articles found that the number-needed-to-biopsy for cutaneous melanoma, which quantifies biopsies resulting in benign diagnoses for every melanoma identified, ranged from 2.2 to 287, with a weighted mean of 15.6 biopsies performed for every melanoma diagnosed worldwide.^1^ This approach is inefficient and may impose significant physical and emotional trauma, especially on those patients who undergo numerous biopsies.

Visual recognition of melanoma is based on morphologic and dermoscopic features.^2^ However, small early-stage melanomas often lack the features associated with more advanced melanomas.^3^ Moreover, visual discrimination of high-grade (severe) dysplasia from banal nevi with low-grade (mild-moderate) dysplasia is not reliable^4^ and poses a clinical problem.^5^ The former cannot be consistently distinguished histologically from melanoma *in situ* and are usually treated as such,^6^ while the latter can safely be monitored and do not require re-excision.^7^ Thus, morphology-based diagnostics lack the ability to identify early molecular changes, underscoring their limitations in early detection.^8^

*In vivo* molecular imaging offers a noninvasive possibility to provide diagnostic molecular information at the point of care to improve early clinical management of pigmented lesions, especially for those in the intermediate “gray area” zone. αvβ3 integrin, which plays a role in neoangiogenesis, lymphangiogenesis, and tumor proliferation, is associated with an aggressive phenotype, making it a promising candidate for molecular imaging.^9^ Integrin αvβ3 drives invasion, migration, and metastasis in breast, pancreatic, and glioblastoma tumors through survival signaling, senescence evasion, and pathways involving MMP-9, Src, and PAK4.^10^, ^11^, ^12^, ^13^ It is expressed in melanoma,^14^ and has also been evaluated as a potential therapeutic target.^15^ Moreover, it is also significantly elevated in melanoma *in situ* compared to benign nevi^14^ and is active during the early stages of melanoma development, when nevi may undergo malignant transformation.^16^^,^^17^ The RGD peptide (Arginine-Glycine-Aspartic acid) motif binds specifically to integrins,^18^ and intravenous administration of radiolabeled RGD peptides has been used for imaging of solid cancers, including melanoma,^19^ breast cancer,^20^ and other advanced solid tumors, such as brain tumors.^21^

The skin fluorescent imaging (SFI) system^22^ utilizes a novel topically applied Cy5.5 fluorescent-labeled RGD peptide (ORL-1), which binds to αvβ3 integrins in the stromal tumor environment of malignant pigmented lesions. In a previous pilot study of 78 lesions scheduled for biopsy, an SFI threshold score of 7 differentiated invasive melanomas from nevi with 100% sensitivity and 95.7% specificity.^22^ Here, we conducted a multicenter, prospective validation study to assess the ability of SFI to differentiate potentially malignant pigmented lesions, including melanoma, in 240 intermediate-risk lesions biopsied from study subjects.

## Methods

### Subjects and lesions

Subjects were enrolled from pigmented lesion clinics at the Huntsman Cancer Institute, and Oregon Health & Science University, and 4 community dermatology practices in California. Adult patients scheduled for biopsy of a clinically suspicious pigmented lesion (<2 cm in diameter and accessible to the imager) provided written consent to participate. The entire clinical lesion was removed after imaging was completed. Subjects who were pregnant or with known sensitivity to fluorescent dyes were excluded. Lesions on oral/mucosal or acral surfaces, convex surfaces, associated with a scar or within proximity to a tattoo, or demonstrated clinical diagnoses of seborrheic keratosis or nonmelanoma skin cancer were excluded. Complete inclusion and exclusion criteria are in Supplementary Methods, available via Mendeley at https://data.mendeley.com/datasets/xntm8w8rbb/5. A total of 11 subjects/lesions were excluded for protocol deviations (*n* = 8) and technical issues (*n* = 3), as detailed in Supplementary Table I, available via Mendeley at https://data.mendeley.com/datasets/xntm8w8rbb/5.

### The SFI system

The integrin-binding ORL-1 peptide (3.3 ng/uL) was applied to the lesion and adjacent skin for 5 minutes and then wiped off, as previously described.^22^ Photographs were obtained with a handheld SFI imager equipped with a modified digital single-lens reflex camera that captures both white light and low-intensity images of fluorescent light emissions across a wavelength range of 350 nm-1100 nm. Images were analyzed using Orlucent Mole Analytics (OMA) software, as previously described,^22^ applying machine learning image processing algorithms to generate a numerical SFI score (ranging from 1-10, to the nearest 0.1 unit) reflecting ORL-1 dye binding to αvβ3 integrin in the lesion environment. A score of 1 represents no αvβ3 integrin binding, and a score of 10 represents significant αvβ3 integrin binding. Additional details of the SFI system have been previously described^22^ and are in Supplementary Methods, available via Mendeley at https://data.mendeley.com/datasets/xntm8w8rbb/5.

### Histopathology

Biopsies were processed under standard conditions, and digital scans of H&E-stained sections were provided to a panel of 3 experienced dermatopathologists each with 17-20 years of experience, who were blinded to the corresponding SFI scores. Independent histopathological evaluation was performed using an updated classification system for melanocytic lesions.^23^ The median consensus diagnosis of the pathology panel for 1 of 5 potential diagnoses (nevus without dysplasia, nevus with low-grade dysplasia, nevus with high-grade dysplasia, melanoma *in situ*, or invasive melanoma) was considered as the histological diagnosis. Lesions were also tagged as high risk (melanoma, melanoma *in situ,* or high-grade dysplasia) or minimal risk (low or no dysplasia) by majority consensus.

### Statistics

Using SFI cutoff scores of 5 and 7, sensitivity, specificity, and other performance metrics were calculated. Prism software (Graphpad, version 10.3.0) was used to compare SFI scores in each diagnostic category and generate a receiver operating characteristic curve. *P* values of <.05 were considered statistically significant.

## Results

### Subjects, lesion diagnoses, and SFI scores

**Fig 1:**
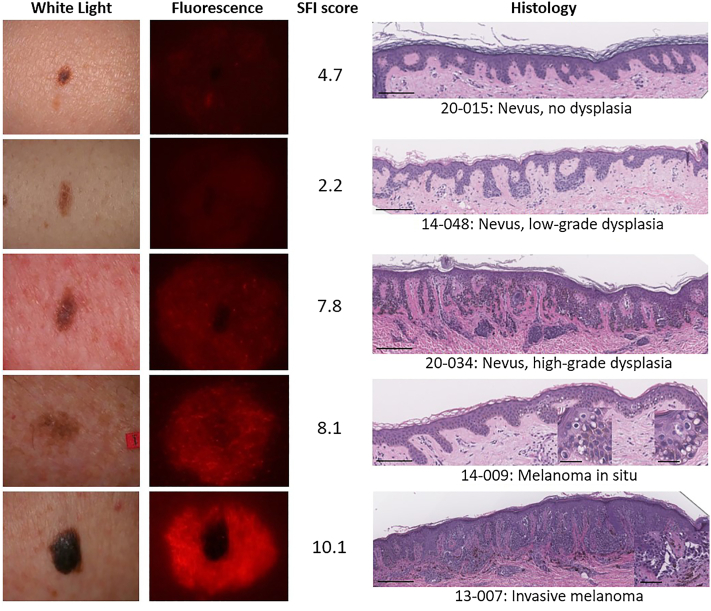
Representative study lesions, reflecting a spectrum from nevi without dysplasia and with low- and high-grade dysplasia to melanoma in situ and invasive melanomas. Shown are images illuminated by *white light* and fluorescence, with corresponding SFI scores and histology. Hyphenated numbers refer to study sites and subject case numbers. Long bars = 400 mm, short bars (inset images) = 70 mm. *SFI*, Skin fluorescence imaging.

**Fig 2:**
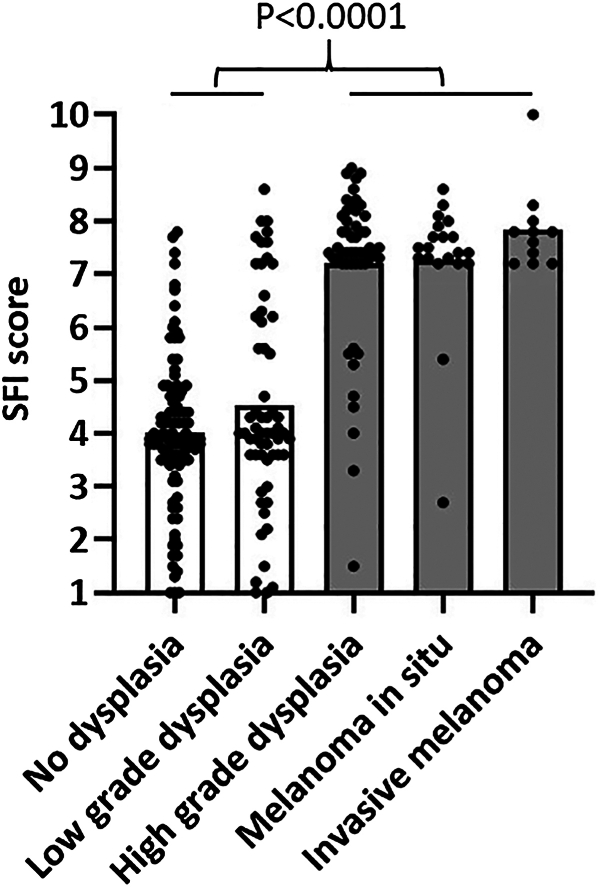
Fluorescence activity increases with lesion dysplasia and is highly associated with melanoma. SFI score is shown for each lesion within each indicated histologic category (*n* = 240 total). Diagnoses were independently determined by consensus from a panel of 3 dermatopathologists. Bars represent mean SFI scores for lesions with no dysplasia (*n* = 99) or low-grade dysplasia (*n* = 59) (open bars) and those demonstrating high-grade dysplasia (*n* = 51), melanoma *in situ* (*n* = 20), or invasive melanoma (*n* = 11) (shaded bars). *P* value from 2-sided t-test. *SFI*, Skin fluorescence imaging.

Subjects (*n* = 240) ranged from 22 to 93 in age (median 62), were predominantly male (60.8%), White (94.6%), and not Hispanic/Latino (95%). Detailed demographic information of the subjects is provided in Supplementary Table II, available via Mendeley at https://data.mendeley.com/datasets/xntm8w8rbb/5. Of the 240 lesions included, their distribution was as follows: 11 invasive melanomas (4.6%), 20 melanoma *in situ* (8.3%), 51 nevi with high-grade dysplasia (21%), 59 nevi with low-grade dysplasia (25%), and 99 lesions without dysplasia (41%). Representative photographic and fluorescent images of lesions, their SFI scores, and histologic presentations are shown in Fig 1. Average Orlucent Mole Analytics scores increased with degree of dysplasia and were highest for melanomas *in situ* and invasive melanomas (Fig 2). Mean SFI scores for high-risk lesions and those with minimal-risk lesions were 7.3 and 4.2, respectively (*P* <.0001, two-tailed t-test).

### Performance of SFI testing

**Fig 3:**
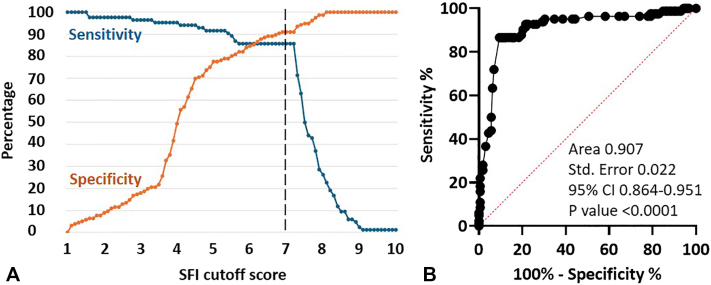
Performance of SFI in distinguishing lesions with no or low-grade dysplasia (*n* = 158) from those demonstrating high-grade dysplasia, melanoma *in situ,* or invasive melanoma (*n* = 82). **A,** Plots of sensitivity and specificity versus SFI cutoff score. Optimal sensitivity and specificity are achieved with a cutoff score of 7 (*dotted line*). **B,** Receiver operating characteristic curve, with associated performance metrics and statistics shown. *SFI*, Skin fluorescence imaging.

Using a cutoff score of 5, the SFI identified high-risk lesions (melanoma, melanoma *in situ,* and high-grade dysplasia) with a sensitivity of 93% and a specificity of 77%. At this cutoff score, the positive and negative predictive values were 68% and 95%, respectively. An SFI cutoff score of 7 distinguished all (11/11, 100%) invasive melanomas from banal nevi, achieving an overall sensitivity of 87% for melanoma, melanoma *in situ,* and high-grade dysplasia with a specificity of 91% (Fig 3, *A*). The positive and negative predictive values for score 7 were 83% and 91%, respectively (Table I). The majority (18 of 20, 90%) of melanoma *in situ* lesions and (42/51, 82%) nevi with high-grade dysplasia had an SFI score above 7 (Table II). By contrast, only 11 of 59 (19%) nevi with low-grade dysplasia and 4 of 99 (4.0%) lesions without dysplasia had an SFI score >7 (Table II). The area under the receiver operating characteristic curve was 0.907 (95% CI 0.864-0.951, *P* < .0001) for discrimination of high-risk lesions from low-risk lesions (Fig 3, *B*). Complete performance metrics at cutoff scores of 5 and 7 are presented in Table I.

**Table I:** Performance metrics of SFI based on cutoff scores of 5 and 7

Diagnosis	Sensitivity (%)	Specificity (%)	PPV (%)	NPV (%)	Accuracy (%)
SFI cutoff = 5					
All (*n* = 240)	93	77	68	95	83
No dysplasia (*n* = 99)		83			
Low-grade dysplasia (*n* = 59)		68			
High-grade dysplasia (*n* = 51)	90				
Melanoma *in situ* (*n* = 20)	95				
Invasive melanoma (*n* = 11)	100				
SFI cutoff = 7					
All (*n* = 240)	87	91	83	91	89
No dysplasia (*n* = 99)		96			
Low-grade dysplasia (*n* = 59)		81			
High-grade dysplasia (*n* = 51)	82				
Melanoma *in situ* (*n* = 20)	90				
Invasive melanoma (*n* = 11)	100				

*NPV*, negative predictive value; *PPV*, positive predictive value; *SFI,* Skin fluorescent imaging.

**Table II:** True and false positives and negatives using SFI score cutoff of 7

SFI score	High-grade dysplasia, melanoma in situ, or invasive melanoma	No dysplasia or low-grade dysplasia
7 or greater	*n* = 71 (true positives)	*n* = 15 (false positives)
<7	*n* = 11 (false negatives)	*N* = 143 (true negatives)

*SFI,* Skin fluorescent imaging.

### Comparison to dermoscopy

Approximately half of the lesions (*n* = 119) were examined by dermoscopy, and in those cases the investigators provided a clinical assessment (low atypia vs moderate and high atypia), which was compared to pathology assessment of low risk (not concerning) and high risk (concerning), and then SFI analysis was performed on the same cases as comparison. Of these 119 cases, 74 were low-grade dysplasia, 30 were high-grade dysplasia, 12 were melanoma *in situ*, and 3 were invasive melanoma by pathological assessment. Both sensitivity and specificity for SFI with a cutoff of 7 were superior to that of dermoscopy alone (89% vs 53% and 94% vs 61%, respectively) for discrimination of low- vs high-risk lesions. Complete performance metrics for dermoscopy alone compared to SFI in these cases are provided in Supplementary Table III, available via Mendeley at https://data.mendeley.com/datasets/xntm8w8rbb/5.

## Discussion

Melanomas constitute a highly aggressive form of skin cancer, accounting for over 90% of skin cancer-related deaths, some subtypes of which are more aggressive than others.^24^ Early detection of lesions with the potential to metastasize is crucial to improving patient outcomes, as the current practice of “when in doubt, cut it out” results in excessive biopsy rates with little impact on reducing mortality rates.

It is estimated that up to half of melanomas reported in the United States may represent overdiagnosis,^25^ referring to diagnosis of melanomas lacking potential to metastasize or cause death. Overdiagnosis results from both increased biopsy rates and the lowering of pathological thresholds^26^ and is characterized by a rising incidence of disease without a corresponding increase in disease-specific mortality over time.^27^^,^^28^ The biopsy rate for pigmented lesions has increased 60-fold over the past few decades.^28^ Furthermore, a population-based study found that melanocytic lesions accounted for 23% of skin biopsies, and 91% of these were benign.^29^ *In vivo* molecular imaging could help identify low-risk lesions that may appear clinically atypical, thus leading to reduction in unnecessary biopsies.

The present clinical trial builds on our prior proof-of-concept pilot study,^22^ evaluating the ability of the SFI system to distinguish between high-risk lesions (eg, nevi with high-grade dysplasia, melanoma *in situ*, or invasive melanoma) and minimal-risk lesions (eg, lesions without dysplasia or low-grade dysplasia) and offers the potential to identify lesions that should be excised as well as those that could be observed despite atypical clinical appearance.

Molecular imaging with SFI enables a quantitative assessment of αvβ3 integrin expression in the melanoma microenvironment, serving as a proxy for the stromal tissue remodeling that occurs during early malignant transformation.^30^^,^^31^ Specifically, αvβ3 integrin expression provides insight into early oncogenic tissue remodeling and angiogenesis associated with melanoma tumor development,^32^ making it a valuable biomarker for assessing early-stage melanoma risk. This approach is likely to offer greater specificity in distinguishing cancerous from noncancerous tissue compared to standard morphologic criteria assessed through visual^33^ and/or dermoscopic^34^ examination. Up to two-third of melanomas may arise *de novo*,^35^ rather than from preexisting nevi, and as such may initially present as amelanotic or bland pigmented macules that often lack clear visual indicators of melanoma until they grow larger.^3^ Future studies will investigate whether the molecular information provided by SFI may provide clinical utility beyond the cellular architecture and structures detected by other in vivo imaging modalities such as dermoscopy and reflectance confocal microscopy.

The SFI system demonstrated high specificity in identifying high-risk lesions that need to be excised and thus has the potential to reduce unnecessary biopsies in at-risk patients while identifying lesions with aggressive potential. In this study, 143 of 158 lesions (91%) that were clinically suspicious for melanoma yet were ultimately benign or showed only low-grade dysplasia had SFI scores below the cutoff of 7 and could have been monitored instead of biopsied. While all invasive melanomas exhibited a score above 7, 2 of 20 (10%) melanoma *in situ,* and 9 of 51 (18%) lesions with high-grade dysplasia did not exhibit integrin expression with a score below 7 (Table II). Presumably, such lesions could be monitored and retested if they enlarged or developed a more suspicious appearance and would yield higher scores upon retesting. On the other hand, only 15 minimal-risk lesions had a score of 7 or greater, representing false positives or exhibiting integrin activity reflecting a potential significant risk (Table II). A more conservative approach could rely on an SFI score of 5 to excise additional melanoma *in situ* and high-grade dysplasia lesions.

SFI may also enhance pathology evaluation by offering a more consistent and objective assessment of lesion dysplasia and risk potential compared to conventional histologic analysis, which is inherently subjective. The interpretation of melanocytic lesions by histopathology often suffers from low interobserver concordance,^36^^,^^37^ reported in some studies to be as low as 70%, potentially leading to both under- and over-diagnosis. SFI may help pathologists who could be overwhelmed with low-risk lesions to concentrate on diagnosing higher-risk lesions.

Dermoscopic assessment can be a critical part of the clinical assessment of melanocytic lesions. Investigators were instructed to employ only the tools they use regularly for clinical evaluation, and lack of application of dermoscopy to all lesions is a limitation of the current study. Dermoscopy was used by some investigators prior to SFI, and a subset analysis of those lesions showed that SFI was superior to dermoscopy alone in discrimination of low-risk from high-risk lesions. Dermoscopy could be included in future studies integrating SFI with established noninvasive methods. Dermoscopy could be easily integrated into the SFI workflow by evaluating a dermoscopic photograph during the short waiting time while the SFI reagents are in contact with the lesion.

In addition to being a noninvasive modality, SFI utilizes few tools and does not require specialized training like other noninvasive imaging modalities, such as reflectance confocal microscopy, to decode images.^38^ While we do not know what the commercial cost of the SFI system will be, it is likely to be far less costly than reflectance confocal microscopy, with limited training and implementation times. Using a handheld imager combined with custom image processing analytics, the SFI system captures and interprets fluorescent αvβ3 integrin patterns associated with early tissue remodeling often not expressed as physical changes, providing rapid (within a few minutes) and actionable results directly at the point of care. Patients with complex nevus phenotypes could have multiple lesions assessed simultaneously. In this context, SFI could be useful in guiding biopsy decision-making and follow-up interval recommendations. Additionally, SFI testing can easily be performed at multiple follow-up visits allowing for repeated assessments over time to evaluate evolving lesions, increasing the likelihood of melanoma detection while mitigating over-reliance on biopsies for histopathological evaluation.

## Conclusion

This clinical trial builds upon findings from a prior proof-of-concept pilot study, supporting the efficacy of SFI as an innovative *in vivo* molecular imaging modality for identifying high-risk lesions at the point of care. By detecting molecular activity associated with malignant potential during early development, SFI offers the potential to reduce unnecessary biopsies while facilitating earlier intervention for high-risk lesions. Ongoing and future studies will help define the most appropriate clinical contexts for its use.

## Conflicts of interest

Drs Manbeck, Shachaf, Majmudar, and Shachaf are employees of Orlucent and have a financial interest in the commercial development of the SFI technology. The other authors have no conflicts of interest to disclose.
